# Glutathione transferases P1/P2 regulate the timing of signaling pathway activations and cell cycle progression during mouse liver regeneration

**DOI:** 10.1038/cddis.2014.562

**Published:** 2015-01-15

**Authors:** J Pajaud, C Ribault, I Ben Mosbah, C Rauch, C Henderson, P Bellaud, C Aninat, P Loyer, F Morel, A Corlu

**Affiliations:** 1 Inserm, UMR 991, Liver, Metabolisms and Cancer, CHU Pontchaillou, Rennes, France; 2 Université de Rennes 1, Faculté de Médecine, Rennes, France; 3 Medical Research Institute, University of Dundee, Ninewells Hospital and Medical School, Dundee, UK; 4 Plateforme Histopathologie H2P2, Biosit, Biogenouest, Université de Rennes 1, Rennes, France

## Abstract

Glutathione transferases (GST) are phase II enzymes catalyzing the detoxification of endogenous noxious compounds and xenobiotics. They also regulate phosphorylation activities of MAPKinases in a catalytic-independent manner. Previous studies have demonstrated the regulation of JNK-dependent pathway by GSTP1/2. Considering the crucial role of JNK in the early steps of the hepatocyte cell cycle, we sought to determine whether GSTP1/2 were essential for hepatocyte proliferation following partial hepatectomy (PH). Using a conventional double knockout mouse model for the *Gstp1* and *Gstp2* genes, we found that the lack of GSTP1/P2 reduced the rate of DNA replication and mitotic index during the first wave of hepatocyte proliferation. The lowered proliferation was associated with the decrease in TNFalpha and IL-6 plasma concentrations, reduced hepatic HGF expression and delayed and/or altered activation of STAT3, JNK and ERK1/2 signaling pathways. In addition, the expression and/or activation of cell cycle regulators such as Cyclin D1, CDK4, E2F1 and MCM7 was postponed demonstrating that the absence of GSTP1/2 delayed the entry into and progression through the G1 phase of the cell cycle and impaired the synchrony of proliferation in hepatocytes following PH. Furthermore, while JNK and its downstream targets c-Jun and ATF2 were activated during the early steps of the liver regeneration in wild-type animals, the constitutively active JNK found in the quiescent liver of *Gstp1/2* knockout mice underwent a decrease in its activity after PH. Transient induction of antioxidant enzymes and nitric oxide synthase were also delayed or repressed during the regenerative response. Altogether our results demonstrate that GSTP1/2 are a critical regulators of hepatocyte proliferation in the initial phases of liver regeneration.

Liver regeneration is a complex and sequential process allowing liver mass restoration after tissue injury. This process is controlled by multiple regulatory pathways that orchestrate both proliferative and hepatoprotective signaling cascades. It has been divided into three distinct phases: the initiation, the promotion or the proliferation step, and the termination.^1^ The initiation, also called priming, corresponds to the activation of the immediate-early response genes by pro-inflammatory cytokines, such as tumor necrosis factor alpha (TNF*α*)^2^ and interleulin-6 (IL-6)^3^ that induces the G0/G1 transition of quiescent hepatocytes. It results in inducing hepatocytes to become sensitive to growth factors and competent for replication. Among the immediate-early response genes, expression of c-Jun and c-Fos is induced to form the transcription factor AP-1 that regulates transcription of genes implicated in cell proliferation or differentiation. Induction of c-Jun requires activation of c-Jun N-terminal kinase (JNK) through the phosphorylation on its Serine 63 and 73 residues. The activation of JNK is therefore an early event that occurs concomitantly to NF-*κ*B activation following TNF*α* signaling.^4, 5^ Among the growth factors involved in the proliferation step, hepatocyte growth factor (HGF), transforming growth factor alpha (TGF*α*) and epidermal growth factor (EGF) induce hepatocytes to override the mitogen restriction point located at 2/3 of G1 phase.^6^ They activate mitogen-activated protein kinase (MAPK) pathways that induce key regulators in late G1 phase such as Cyclin D's and cyclin-dependent-kinases 4/6 (CDKs), which have a critical role in the control of the G1/S transition and the commitment to DNA replication.^7, 8, 9, 10, 11^ Gene-deficient knockout mouse models have not only confirmed the importance of cytokines and growth factors but also revealed the role of metabolic networks in integrating regenerative response with maintenance of hepatic function. Despite numerous studies during the past decades, cell-extrinsic and -intrinsic molecular mechanisms governing the dynamic metabolic changes in hepatocytes after partial hepatectomy (PH) still remain incompletely understood.

Hemodynamic changes induced by PH and pro-inflammatory cytokines are also known to cause alteration of redox potential affecting the stress-activated signaling cascades. Glutathione transferase (GST) phase II detoxification enzymes belong to a multigenic family with seven classes (alpha, mu, pi, omega, sigma, theta, zeta). They are involved in various functions, including detoxification, biosynthesis and cell signaling.^12, 13, 14, 15^ Conjugation activities of GSTs result in cell protection by eliminating noxious compounds, by protecting proteins, lipids or DNA against reactive species and by metabolizing molecules, such as prostaglandins^16^ and 4-hydroxy-2-nonenal.^17^ GSTs catalyze glutathionylation or S-glutathionylation, a reversible posttranslational modification regulating protein function, which is significantly promoted by reactive nitrogen species and reactive oxygen species (ROS). GSTs also have important roles in regulating signaling pathways in a catalytic-independent manner through direct interaction with proteins such as protein kinases to modulate their phosphorylation activities. For example, GSTA1^18^ and mGSTA4^19^ bind to JNK, and GSTM1 interacts with apoptosis signal-regulating kinase 1 (ASK1), the kinase transducing JNK signaling, to inhibit the apoptotic pathways.^20^ During oxidative stress in mouse hepatocytes, it was shown that JNK is required for the induction of GSTA4 leading to the formation of mGSTA4/JNK complexes that might regulate JNK activity.^19^ GSTM1, GSTA1 and GSTP1 also have a key role in the regulation of ASK1 phosphorylation activity in rat hepatocytes.^21^ In fibroblasts 3T3/4A, GSTP1/JNK interaction occurring in the absence of stress stimuli also inhibits JNK activity and c-Jun phosphorylation.^22^ More recently, in osteosarcoma cells, GSTP1 has been shown to interfere not only with JNK pathway but also with tumor necrosis factor receptor-associated factor 2 (TRAF2) signaling resulting in the inhibition of P38 and JNK phosphorylation. The dissociation of the complexes leads to ASK1 activation and prolonged activation of P38 and JNK that cause cell growth arrest by increasing the expression of the CDK inhibitor P21^CIP1^ and apoptosis.^23^

Nowadays, there is no report on the possible involvement of GSTP1 in the regulation of liver regeneration although the crucial role of JNK pathway in the entry into and progression through G1 phase of the cell cycle in hepatocyte is well established.^5, 24^ Using a conventional double knockout mouse model for the *GstP1* and *GstP2* genes,^25^ we investigated for the first time the impact of the absence of GSP1/P2 on liver regeneration after two-third PH. Our data demonstrate that GSTP1/P2 contribute to the finely tuned activation levels of proliferation signaling pathways and to the downstream expression of cell cycle regulators in order to achieve the proper proliferation rate of hepatocytes and the cell cycle synchrony during liver regeneration.

## Results

### Expression of GSTP1/P2 increases in regenerating liver

**Figure 1:**
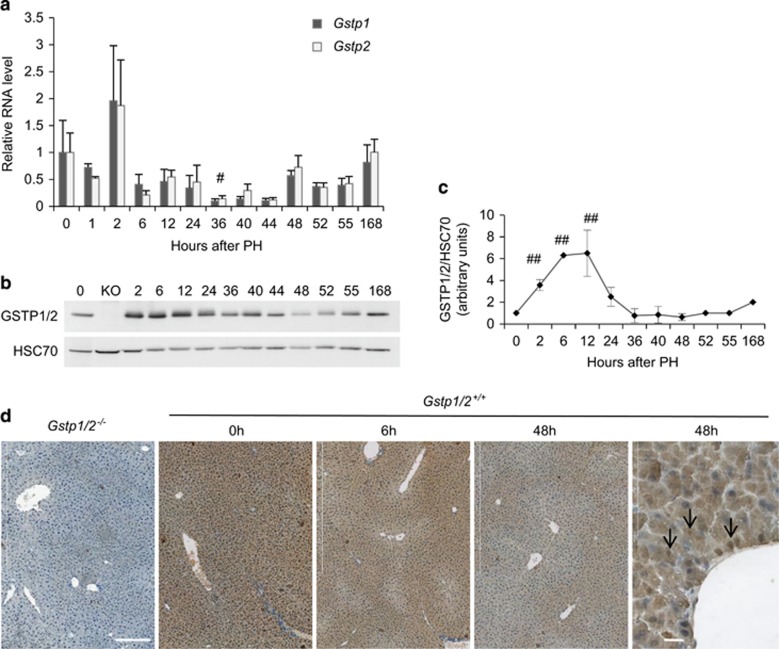
Expression and hepatic localization of GSTP1/2 after PH in WT mice. (**a**) mRNA levels of hepatic *Gstp1* (grey bar) and *Gstp2* (white bar) were measured by RT-qPCR at the indicated times after PH. Results are expressed as fold induction compared with the control liver arbitrarily set at 1 and as mean±S.E.M. (*n*=3–7 mice/group/time point). ^#^*P*≤0.05, ^##^*P*≤0.01, time point after PH *versus* normal livers. (**b**) Pool of total proteins from different mice were used for western blotting analyses of GSTP1/2 expression in the livers of *Gstp1/2*^*+/+*^ mice at the indicated times after PH. HSC70 is used as a loading control. (**c**) Densitometric analysis of the western blotting results of GSTP1/2 obtained from different mice (*n*=3). (**d**) Immunolocalization of GSTP1/2 in the WT livers prior to PH and at 6 and 48 h after PH. Arrows indicate some stained nuclei. The livers of *Gstp1/2*^*−/−*^ mice were used as negative controls. Bars: 200 or 20 *μ*m

Following PH, *Gstp1* and *p2* mRNA levels in regenerating livers increased at 2 h when compared with the normal liver and then dropped at 36 h to levels below to those found in the normal liver (Figure 1a). In agreement, the protein amounts augmented rapidly after PH before decreasing in a time-dependent manner until 48 h (Figure 1b). GSTP1/P2 immunodetection showed a homogeneous staining across the hepatic lobule in the normal liver while the expression appeared mainly concentrated in periportal hepatocytes at 6 and 48 h in regenerating livers (Figure 1c). Consistent with western blotting results, GSTP1/P2 staining was greatly diminished at 48 h post-PH. Of note, GSTP1/P2 were found in the nucleus of some regenerating hepatocytes. No labeling was detected in the *Gstp1/2*^*−/−*^ mouse livers.

### Loss of *Gstp1/2* does not modify hepatocyte survival following PH

After PH, postoperative survival was similar for wild-type (WT) and *Gstp1/2*^*−/−*^ mice when the gallbladder was kept intact. Indeed, the number and extent of bile infarcts increased in *Gstp1/2*^*−/−*^ mice compared with WT animals when gallbladder alterations occurred (data not shown).

**Figure 2:**
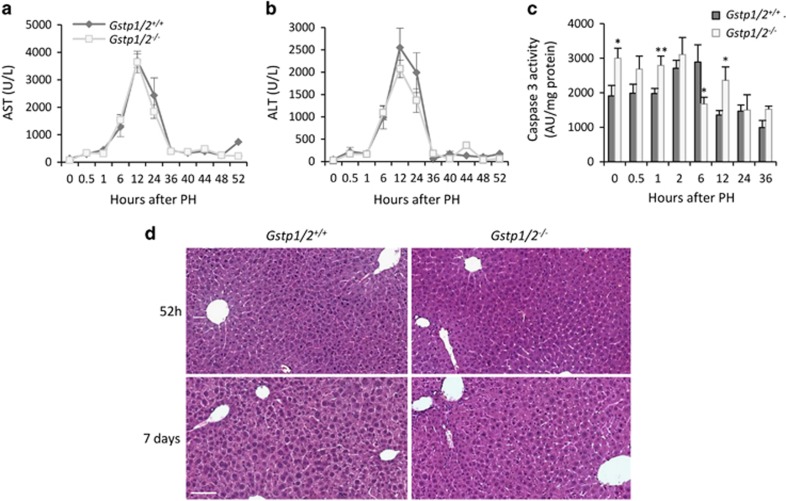
Transaminase levels and cell death in *Gstp1/2*^*−/−*^ mice after PH. Serum levels of (**a**) AST and (**b**) ALT in *Gstp1/2*^*+/+*^ and *Gstp1/2*^*−/−*^ mice at the indicated times after PH. (**c**) Caspase 3 activity in the liver homogenates of *Gstp1/2*^*+/+*^ and *Gstp1/2*^*−/−*^ mice at the indicated times after PH. Results were expressed as mean±S.E.M. (*n*=3–7 mice/group/time point). *P≤0.05, **P≤0.01, *Gstp1/2*^*−/−*^ *versus Gstp1/2*^*+/+*^ mice. (**d**) Histology of the livers from *Gstp1/2*^*+/+*^ and *Gstp1/2*^*−/−*^ mice at 52 h and 7 days after PH. Bar: 100 *μ*m

In the absence of gallbladder alterations, histological analysis of regenerating livers did not reveal abnormalities or signs of accelerated inflammatory reaction in *Gstp1/2*^*−/−*^ mice (Figure 2). Serum levels of aspartate aminotransferase (AST) and alanine aminotransferase (ALT) peaked at 12 h in both mice genotypes (Figures 2a and b). Caspase 3 activity increased at 6 h in WT mice before returning to basal level at 12 h (Figure 2c). In *Gstp1/2*^*−/−*^ mice, basal level of Caspase 3 activity was significantly higher than in the WT normal livers, and this activity did not show any increase over the time course performed. Blood levels of triglycerides, urea and bilirubin were not significantly affected and indicated that the overall liver function remained efficient in both mice genotypes (data not shown).

### Invalidation of *Gstp1/2* impairs hepatocyte proliferation

**Figure 3:**
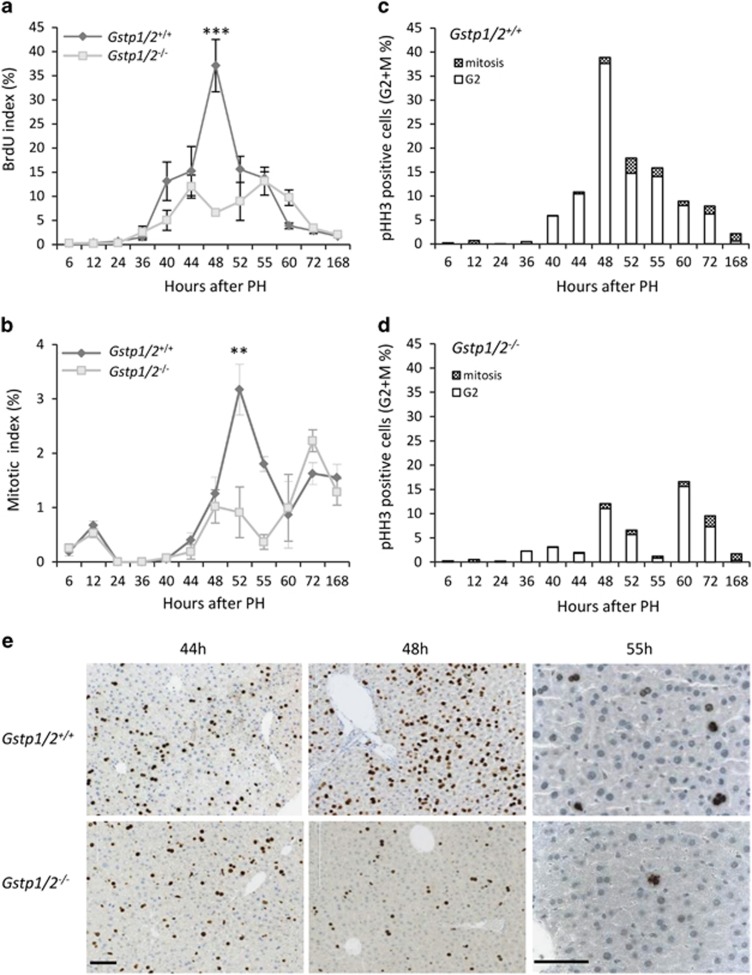
Defective hepatocyte proliferation in *Gstp1/2*^*−/−*^ mice after PH. The percentage of (**a**) BrdU-positive hepatocytes and of (**b**) hepatocytes in mitosis in the *Gstp1/2*^*+/+*^ and *Gstp1/2*^*−/−*^ mouse livers at the indicated times after PH. The percentage of phospho-histone H3 (pHH3)-positive hepatocytes in G2/M phase in the (**c**) *Gstp1/2*^*+/+*^ and (**d**) *Gstp1/2*^*−/−*^ livers at the indicated times after PH. Quantification was performed by analyzing at least six microscope fields ( × 20) per mice. Results were expressed as mean±S.E.M. (*n*=3–7 mice/group/time point). **P≤0.01, ***P≤0.001, *Gstp1/2*^*−/−*^ *versus* WT mice. (**e**) Immunolocalization of BrdU or of pHH3 at the indicated times after PH, Bars: 100 *μ*m

In WT mice, the percentage of 5-bromo-2'-deoxyuridine (BrdU)-positive hepatocytes increased at 40 h to reach a peak 48 h after PH. Then hepatocyte DNA synthesis returned to basal level at 3 days. In the livers of *Gstp1/2*^*−/−*^ mice, DNA synthesis also began at 40 h but did not show any sharp increase during the 72-h time post-PH. In mutant mice, only 6.58±1.71% of hepatocytes were BrdU-positive at 48 h *versus* 37.07±10.8% in the WT livers (Figures 3a and e). Mitotic index (Figure 3b) and the percentage of phosphorylated histone H3-positive hepatocytes (Figures 3c and d) further evidenced a significant decrease in the proliferation rate in the *Gstp1/2*^*−/−*^ livers compared with the WT livers. At 52 and 55 h, the *Gstp1/2*^*−/−*^ livers displayed an ~3-fold decrease in the cell numbers in G2 and M phases compared with their control counterparts. Altogether, these data indicate that lack of GSTP1/P2 significantly impaired the partially synchronized first wave of hepatocyte proliferation without abolishing totally the process of liver regeneration.

### Loss of *Gstp1/2* alters the expression timing of cell cycle regulator expression

**Figure 4:**
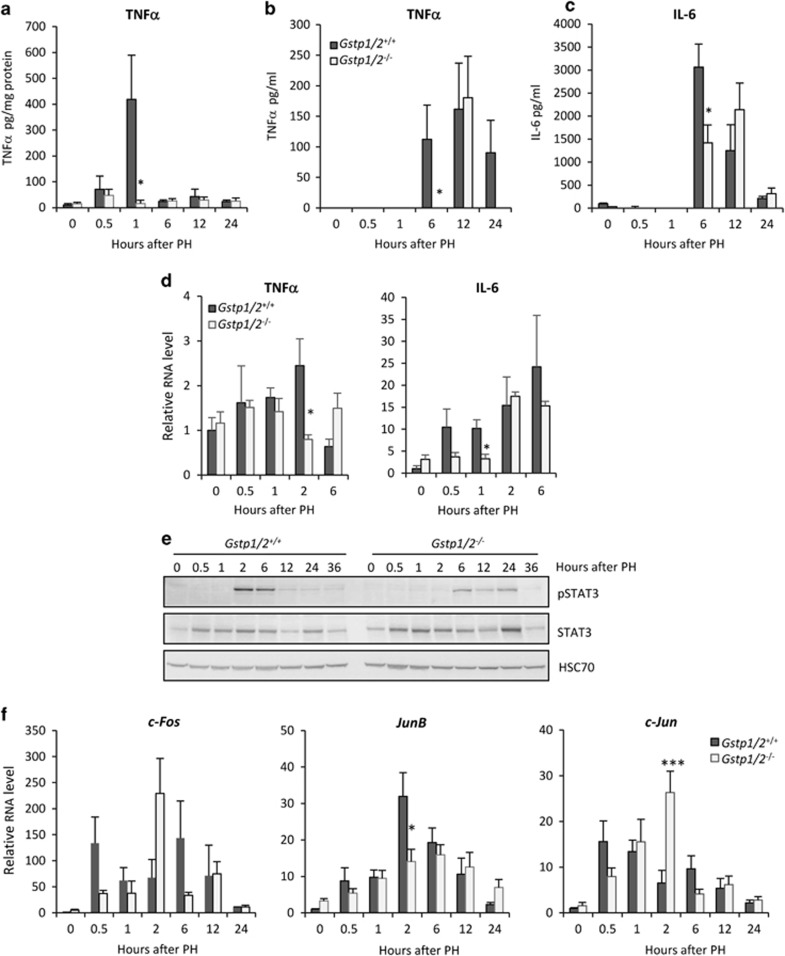
Alteration of the hepatocyte ‘priming' after PH in *Gstp1/2*^*−/−*^ mice. (**a**) Intrahepatic levels of TNF*α* and the serum levels of (**b**) TNF*α* and (**c**) IL-6 in *Gstp1/2*^*+/+*^ and *Gstp1/2*^*−/−*^ mice at indicated times after PH. (**d**) mRNA levels of hepatic *Tnfα* and *Il-6* were measured by RT-qPCR at the indicated times after PH. (**e**) Pool of total proteins from three mice per time point were used for western blotting analysis of STAT3 activation in the *Gstp1/2*^*+/+*^ and *Gstp1/2*^*−/−*^ livers. HSC70 is used as a loading control. (**f**) mRNA levels of *c-Jun*, *c-Fos* and *JunB* proto-oncogenes by qPCR in *Gstp1/2*^*+/+*^ and *Gstp1/2*^*−/−*^ mice at the indicated times after PH. Results are expressed as fold induction compared with the *Gstp1/2*^*+/+*^control liver arbitrarily set at 1 and as mean±S.E.M. (*n*=3–7 mice/group/time point). *P≤0.05, ***P≤0.001, *Gstp1/2*^*−/−*^ *versus Gstp1/2*^*+/+*^mice

**Figure 5:**
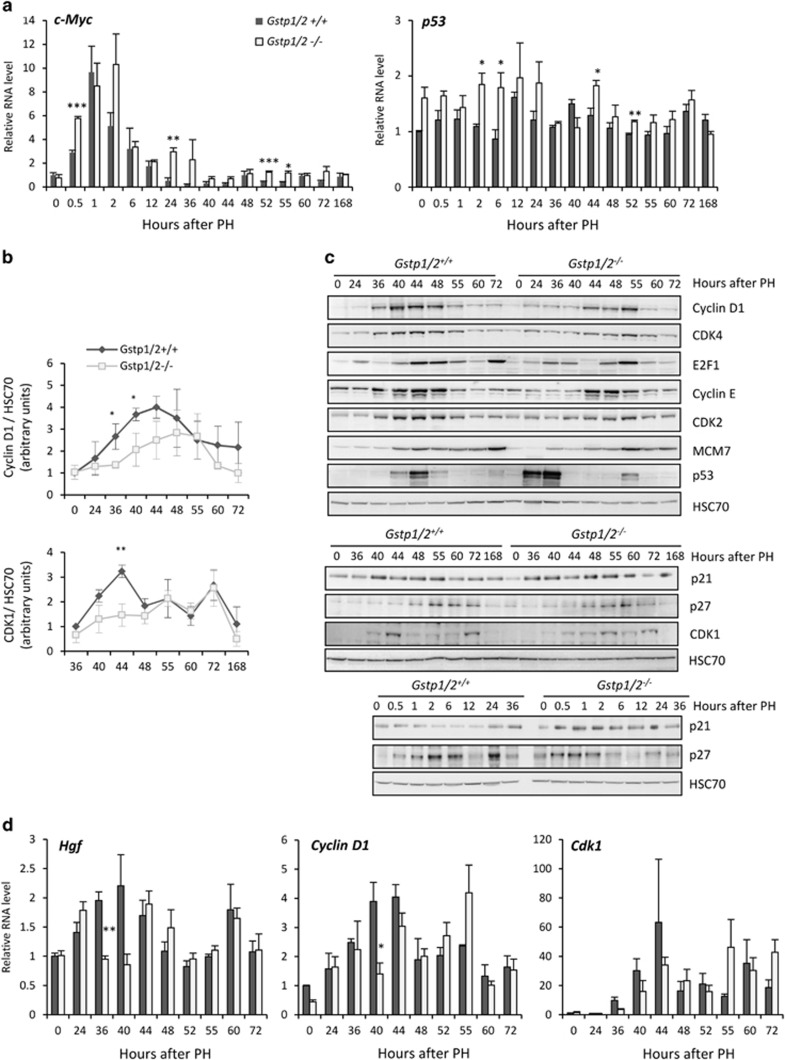
Differential mRNA and protein expression profiles of cell cycle regulators in the livers of *Gstp1/2*^*+/+*^ and *Gstp1/2*^*−/−*^ mice after PH. (**a**) mRNA levels of *c-Myc* and *p53* in the *Gstp1/2*^*+/+*^ and *Gstp1/2*^*−/−*^ livers at the indicated times after PH. (**b**) Densitometry analysis of the western blotting results of CyclinD1 and CDK1 obtained from different mice at the indicated times (*n*=3). (**c**) Pool of total proteins were used for western blotting analysis of cell cycle regulators in the livers of *Gstp1/2*^*+/+*^ and *Gstp1/2*^*−/−*^mice at the indicated times after PH. HSC70 is used as a loading control. (**d**) mRNA levels of *Hgf*, *Cyclin D1* and *Cdk1* in the livers of *Gstp1/2*^*+/+*^ and *Gstp1/2*^*−/−*^ mice at the indicated times after PH. Results are expressed as fold induction compared with the *Gstp1/2*^*+/+*^ control liver arbitrarily set at 1 and as mean±S.E.M. (*n*=3–7 mice/group/time point). *P≤0.05, **P≤0.01, ***P≤0.001, *Gstp1/2*^*−/−*^ *versus Gstp1/2*^*+/+*^ mice

To evaluate the effect of *Gstp1/2* deletion on hepatocyte cycle progression, we analyzed the expression of main regulators involved in G0/G1 transition. As expected, intrahepatic protein and RNA levels as well as serum levels of TNF*α* rapidly increased at 1, 2 and 6 h post-PH in WT mice, respectively (Figures 4a, b and d). In parallel, liver RNA and serum levels of IL-6 augmented at 0.5–1 and 6 h post-PH, respectively (Figures 4c and d). In contrast, the production of TNF*α* and IL-6 was delayed in *Gstp1/2*^*−/−*^ mice, and their serum concentration peaked after only 12 h. The changes in IL-6 secretion correlated with the delayed phosphorylation of STAT3 in the *Gstp1/2*^*−/−*^ livers (Figure 4e) and a postponed and/or reduced expression of *c-Fos*, *Jun B* and *c-Jun* mRNA levels during the first 24 h post-PH (Figure 4f). In contrast, induction of *c-Myc* mRNAs occurred earlier, and their levels remained higher at several time points in the *Gstp1/2*^*−/−*^ regenerating livers compared with the WT control livers (Figure 5a). *P53* mRNA levels were also slightly higher during the first 24 h post-PH in *Gstp1/2*^*−/−*^ mice (Figure 5a).

We further studied the time course of the expression of cell cycle regulators involved in the mid-late G1 phase, the G1/S transition and the DNA replication. At 36 and 40 h, in mid-late G1 phase, the hepatic mRNA levels of *Hgf* appeared significantly lower in *Gstp1/2*^*−/−*^ along with a limited induction of *Cyclin D1* mRNA levels at 40 h compared with their strong increase in the WT livers (Figure 5d). In agreement, the amount of Cyclin D1 protein was strongly induced in the WT regenerating livers from 36 to 48 h prior the onset of DNA synthesis while its expression gradually increased in a time-dependent manner leading to the maximal levels after only 55 h in the *Gstp1/2*^*−/−*^ livers (Figures 5b and c). Similar patterns of expression were observed for CDK4 with a delayed induction in *Gstp1/2*^*−/−*^. The expression of the proteins E2F1, Cyclin E, CDK2 and MCM7, which characterize the progression in late G1 phase and the commitment to DNA replication, was also postponed in *Gstp1/2*^*−/−*^ (Figure 5c). Importantly, the expression of tumor suppressor protein P53 occurred earlier in the *Gstp1/2*^*−/−*^ regenerating livers with a sharp induction at 24 and 36 h while in the WT livers its expression mainly detected at 40 and 44 h was much lower. The levels of cell cycle inhibitors P21^CIP1^ and P27^Kip1^ were also altered in *Gstp1/2*^*−/−*^ reinforcing the conclusion of an impaired progression in G1 phase in mutant mice (Figure 5c). High levels of P21^CIP1^ were maintained during all the regeneration period in *Gstp1/2*^*−/−*^ mice, including the early time points, whereas P21^CIP1^ amounts increased only at 40 h in the WT livers. The basal protein levels of P27^Kip1^, high in *Gstp1/2*^*−/−*^ mice, decreased rapidly from 2 up to 36 h, whereas its expression low in the normal WT liver increased at 6 and 24 h. Also, P27^Kip1^ expression was similar in both mice genotypes. Although, the induction of the *Cdk1* mRNA expression was found relatively similar in both mice genotypes (Figure 5d), CDK1 protein, which is known to be expressed during DNA replication and progression in G2 and M phases, was transiently induced at 40 and 44 h in the WT livers while its expression increased progressively with maximal levels between 55 and 72 h in mutant mice (Figures 5b and c).

### Activation of JNK and extracellular signal-regulated kinase (ERK) signaling pathways are altered in *Gstp1/2*^*−/−*^ mice following PH

**Figure 6:**
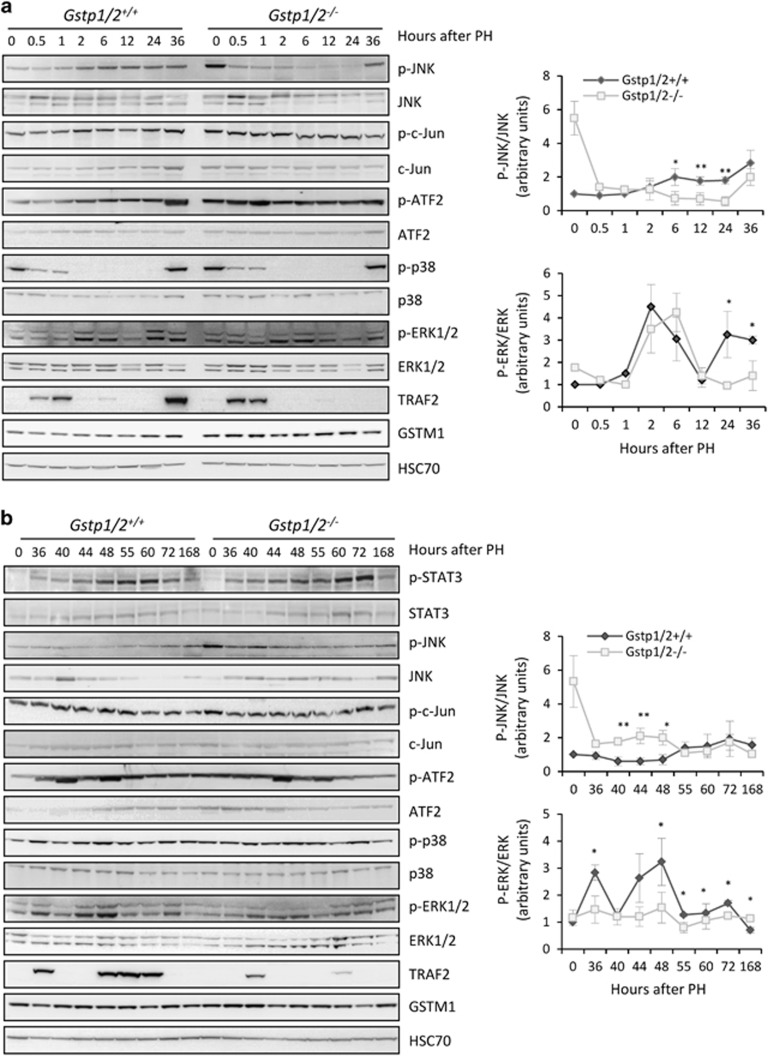
Cell signaling protein expression and/or activation in *Gstp1/2*^*+/+*^ and *Gstp1/2*^*−/−*^mice after PH. Pool of total proteins from three mice were used for western blotting analyses of JNK, c-Jun, ATF2, p38, ERK1/2 phosphorylation and TRAF2 and GSTM1 expression in *Gstp1/2*^*+/+*^ and *Gstp1/2*^*−/−*^mice at the indicated times after PH (**a**) early time points and (**b**) late time points. HSC70 is used as a loading control. (**c**) Densitometry analysis of the western blotting results of p-JNK and p-ERK obtained from different mice at the indicated times (*n*=3). *P≤0.05, **P≤0.01, *Gstp1/2^−/−^ versus Gstp1/2^+/+^* mice

To address the molecular mechanisms inducing the delayed liver regeneration and the alteration of cell cycle regulators expression in *Gstp1/2*^*−/−*^ mice, we investigated the activation of signaling pathways. As previously reported,^26^ we observed a constitutive activation of JNK pathway in the *Gstp1/2*^*−/−*^ mouse livers prior to PH (Figure 6a). Indeed, the phosphorylation of JNK and its downstream substrates c-Jun and activating transcription factor 2 (ATF2) were higher in the livers of *Gstp1/2*^*−/−*^ mice than in the WT livers. Following PH, the levels of p-JNK and p-c-Jun decreased progressively until 24 h while that of p-ATF2 remained stable (Figure 6a). Levels of p-JNK increased only at later time points (Figure 6b). Conversely, in WT mice, JNK pathway was activated from 2 to 40 h as evidenced by the enhanced phosphorylation levels of JNK, c-Jun and ATF2 (Figure 6). At later time points (40–168 h), p-JNK kept increasing, whereas the levels of p-c-Jun and p-ATF2 decreased.

Regarding P38 activation, similar profiles were obtained in WT and *Gstp1/2*^*−/−*^ mice. Phospho-P38 expressed in the quiescent liver underwent a rapid but transient dephosphorylation lasting 24 h (Figure 6a). Then induction of p38 activation was observed 36 h after PH and remained stable until 168 h (Figure 6b). ERK1/2 activation occurred successively three times in the liver of WT mice at 2–6, 24–36 and 44–48 h after PH (Figures 6a and b). In *Gstp1/2*^*−/−*^ mice, a similar induction of ERK1/2 phosphorylation was observed at 2–6 h. In contrast, ERK1/2 phosphorylation was not induced at 24–36 h and was moderately increased at 44–48 h in mutant mice. TRAF2 transiently induced at 0.5–1 h in both mice genotypes was re-expressed in WT at 36 and 48–60 h after PH, whereas it was barely detectable at 40 and 60 h in mutant mice.

### Expression of stress response genes is delayed in *Gstp1/2*^*−/−*^ mice after PH

**Figure 7:**
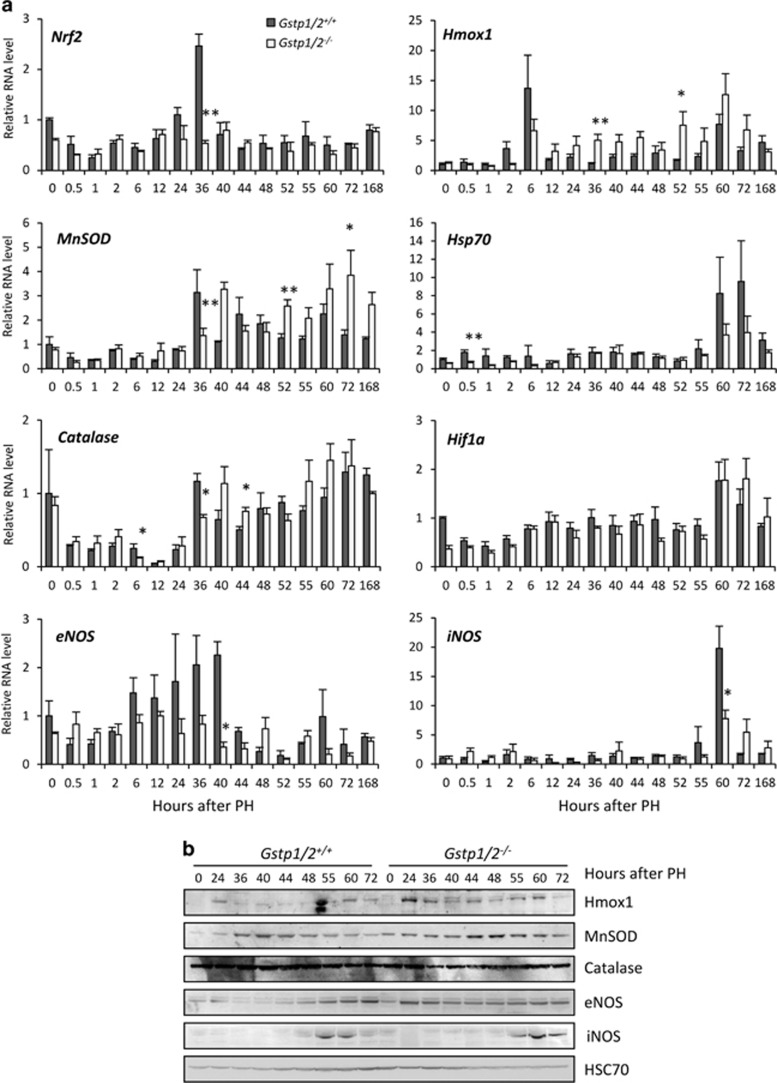
Differential mRNA expression of antioxidant enzymes in the livers of *Gstp1/2*^*+/+*^ and *Gstp1/2*^*−/−*^mice after PH. (**a**) mRNA of *Nrf2*, *MnSOD*, *catalase*, *Hmox-1*, *Hsp70*, *Hif1α*, *eNOS* and *iNOS* expression in *Gstp1/2*^*+/+*^ and *Gstp1/2*^*−/−*^ mice at the indicated times after PH. Results are expressed as fold induction compared with the *Gstp1/2*^*+/+*^ control liver arbitrarily set at 1 and as mean±S.E.M. (*n*=3–7 mice/group/time point). *P≤0.05, **P≤0.01, *Gstp1/2*^*−/−*^ *versus Gstp1/2*^*+/+*^ mice. (**b**) Pool of total proteins from three mice per time point were used for western blotting analysis of eNOS and iNOS in *Gstp1/2*^*+/+*^ and *Gstp1/2*^*−/−*^mice at the indicated times after PH. HSC70 is used as a loading control

**Figure 8:**
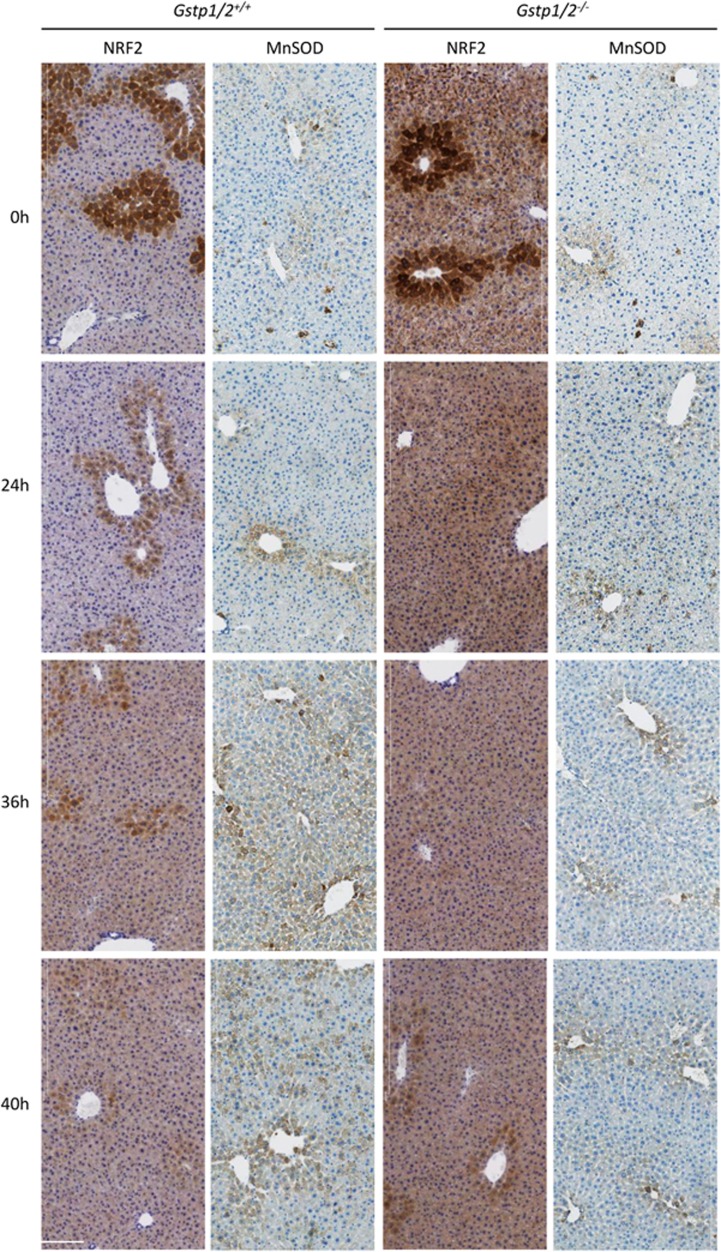
Hepatic localization of NRF2 and MnSOD after PH in *Gstp1/2*^*+/+*^ and *Gstp1/2*^*−/−*^ mice. Immunolocalization of NRF2 and MnSOD in the *Gstp1/2*^*+/+*^ and *Gstp1/2*^*−/−*^ livers prior to PH (0 h) and at 24, 36 and 48 h after PH. Bars: 200 *μ*m

ROS modulate MAPK activation such as JNK, which is essential for regulating the intracellular redox status and the balance between cell proliferation and cell death. Among the enzymes contributing to the balance of the redox system, GSTP1/P2 catalyze the elimination of electrophilic oxidants. We therefore studied the expression of enzymes involved in cell defense mechanisms. After PH in WT mice, RNA levels of *Nrf2* increased at 36 h concomitantly with the induction of its two target genes, the mitochondrial superoxide dismutase (MnSOD) and the catalase both at the RNA (Figure 7a) and protein levels (Figure 7b). Changes in NRF2 and MnSOD liver distribution were also evidenced. Mainly present in the perivenous areas, NRF2 and MnSOD expressions became more diffuse across the hepatic lobule at 24 and 36 h post-PH (Figure 8). Regarding heme oxygenase 1 (Hmox1), its expression both at the RNA and protein levels showed a biphasic induction at 6–24 and 60 h (Figures 7a and b). In mutant mice, the expression profiles of *Nrf2*, *MnSOD*, *catalase* and *Hmox1* were different. No increase in *Nrf2* RNA levels were detected during the regenerative response, whereas RNA and protein amounts of *MnSOD* and *catalase* increased but only at 40–44 h and at late time points. Consistent with these changes, NRF2 and MnSOD staining located mainly in the perivenous areas, and also in the sinusoids for NRF2 in the *Gstp1/2*^*−/−*^ livers, became more diffuse in the hepatic lobule from 40 h post-PH (Figure 8). In contrast, *Hmox1* RNA and protein levels progressively increased to become higher than in WT mice at ~36 and 52 h (Figure 7a).

Expression of *Hsp70* involved in correct folding and elimination of misfolded proteins was similar in both mice genotypes. *Hif1α* mRNA expression did not differ between the control and mutant mice although the initial level of *Hif1α* expression was higher in the WT livers than in the *Gstp1/2*^*−/−*^ livers (Figure 7a).

Interestingly, the expression of the nitric oxide synthases, eNOS and iNOS, involved in NO production and hemodynamic changes was also altered in *Gstp1/2*^*−/−*^ mice. In WT mice, *eNOS* RNA levels increased from 6 to 40 h post-PH. This increase correlated with the accumulation of eNOS protein from 24 to 72 h (Figure 7b). RNA and protein levels of iNOS increased only at late stages of the regeneration, 55–60 h post-PH. In contrast, although the RNA levels of *eNOS* remained lower in the *Gstp1/2*^*−/−*^ livers than those found in the WT livers, the protein was highly expressed at least from 24 to 72 h post-PH in the *Gstp1/2*^*−/−*^ livers. In addition, the maximum levels of *iNOS* mRNA also found at 60 h were significantly lower than those in the WT livers, and the protein was detected later at 60–72 h post-PH.

## Discussion

Liver regeneration is characterized by a precise temporal pattern of events that regulate the entry into and progression through the cell cycle, including the progression beyond the mitogen-dependent restriction point in late G1 phase, the G1/S transition and mitosis.^27, 28^ Our laboratory has previously shown the inductions of GSTA1, GSTA4, GSTM1 and GSTP1/P2 during mouse liver regeneration.^29^ Here we confirmed the early induction of GSTP1/P2 following PH and evidenced for the first time that this induction was associated with a predominant periportal intra-lobular localization. This portal zonation reinforced the hypothesis that GSTP1/P2 could contribute to liver regeneration as hepatocyte proliferation begins in the periportal areas^30^ and that gene inductions linked to cell proliferation mainly occurs in these areas.^31^ Using a conventional double knockout mouse model for the *Gstp1* and *p2* genes, we show that GSTP1/P2 is required for the activation of signaling pathways involved in liver regeneration. In WT mice, the first wave of hepatocyte proliferation is completed within 72 h after two-third hepatectomy. In *Gstp1/2*^*−/−*^ mice, the lack of GSTP1/P2 strongly modified the chronology of the induction of cell cycle regulators and the magnitude of the proliferative response. The secretions of TNF*α* and IL-6, the priming agents regulating the entry into G1 phase, were delayed leading to a postponed induction of the immediate-early genes such as *c-Fos* and *Jun-B* as well as a late and reduced activation of STAT3. Then the increase in *Hgf* and *Cyclin D1* RNA amounts, occurring at 36 and 40 h in WT mice, respectively, appeared only at 40 and 44 h in *Gstp1/2*^*−/−*^ mice. The delay in *Hgf* and *Cyclin D1* expression also coincided with differences in the kinetics of ERK1/2 phosphorylation between the two mice genotypes, suggesting that growth factor stimulation required for hepatocyte progression up to G1/S boundary was also altered.^32, 33^ In both mice, the first activation of ERK1/2 signaling pathway occurred in early G1. However, the second burst of activation happening in mid-late G1 phase was lowered in *Gstp1/2*^*−/−*^ mice, and the downstream inductions of Cyclin D1 and its catalytic partner CDK4 were delayed. This delayed induction of Cyclin D1 and CDK4 partners controlling the progression in late G1 phase led to alteration in E2F1 accumulation that allows the coordination of gene transcription necessary for DNA replication. Accordingly, the expression of Cyclins E and A, two E2F1-target genes, and the induction of MCM7, CDK2 and CDK1 were postponed. Together, these delayed and/or reduced inductions of crucial cell cycle regulators in early and mid-late G1 demonstrate that the G0/G1 transition, the progression beyond the mitogen-dependent restriction point and the G1/S transition were significantly altered by the absence of GSTP1/2. Finally, the third burst of ERK1/2 activation associated with G2/M progression^34^ was also greatly diminished and associated with a reduced number of cells replicating DNA and progressing in G2 and M phases.

Several other MAPKinases, including P38 and JNK, also drive specific cell cycle responses following extracellular stimuli. During liver regeneration, JNK and c-Jun activations are important early events. Indeed, genetic invalidation of JNK^5, 35^ or activation of JNK prior to PH^36^ decreases or improves hepatocyte proliferation, respectively. As previously reported,^26^ we found that JNK is constitutively activated in the control livers of *Gstp1/2*^*−/−*^ mice as demonstrated by the high amounts of p-JNK. Unexpectedly, this activation was rapidly reduced after PH in *Gstp1/2*^*−/−*^ mice. This diminution of JNK activity was correlated to increased GSTM1 protein levels in the early phase of liver regeneration in the *Gstp1/2*^*−/−*^livers. Indeed, GSTM1 inhibits the activity of the upstream regulator of JNK, the protein kinase ASK1. Concomitantly to the decrease in p-JNK levels in *Gstp1/2*^*−/−*^ mice, we observed the inactivation of its downstream substrates c-Jun and ATF2, whereas their phosphorylation levels increased in WT mice. As the c-Jun/ATF2 heterodimer induces transcription of many genes such as *Cyclin D1*, *Cyclin A*, *c-Jun* and *Atf3*,^37^ changes in JNK activation most likely contributes along with ERK1/2 signaling alterations to the delay in G1-to-S phase progression in *Gstp1/2*^*−/−*^ mice. Given that the transcription factor ATF3 induces P21^CIP^ expression while high c-Jun activity represses P53-mediated P21^CIP^ induction,^38, 39^ the precise c-Jun/ATF2 and ERK1/2 activations regulate the transient increase in p21^CIP^ protein amounts required to stabilize CyclinD/CDK4/6 complexes from 36 to 40 h in WT mice. In contrast, in *Gstp1/2*^*−/−*^ mice, lower activations of JNK, c-Jun/ATF2 and ERK1/2 trigger higher and longer expression of P53 and P21^CIP^ that may contribute to the alterations of hepatocyte proliferation.

Considering the alterations of JNK and ERK1/2 activations in *Gstp1/2*^*−/−*^ mice, we have also investigated the expression of genes induced by the cellular redox status and hemodynamic changes that arise after liver resection.^40, 41^ To avoid cellular and DNA damages, ROS concentration is tightly regulated during liver regeneration, and this process is achieved at least in part by the cytoprotective transcription factor Nrf2. In agreement with the lower MAPK activation in *Gstp1/2*^*−/−*^ mice, the transient increase in *Nrf2* mRNA levels at the time of MAPK activation (36 h) was not observed. However, the ROS-detoxifying Nrf2 target genes, MnSOD and catalase, were eventually induced but with a significant delayed expression compared with the WT mice. Interestingly, *Hmox-1* RNA levels, not increased at 6 h like in WT mice, were progressively augmented later and may have a protective role during liver regeneration by modulating MnSOD and catalase expression. As previously reported, Hmox-1 expression could result from NO release in response to shear stress.^42^ Indeed, the high eNOS protein levels detected from 24 to 72 h in *Gstp1/2*^*−/−*^ mice suggests an enhanced NO production during this period of time. In parallel, the higher but transient P53 expression in *Gstp1/2*^*−/−*^ mice could contribute to the maintenance of the redox homeostasis and cell survival as well as the early increase in GSTM1 protein levels that blocks the TRAF2/ASK1 signaling pathway. Accordingly, higher levels of caspase 3 activities were detected in the liver of *Gstp1/2*^*−/−*^ mice, and massive cell death was induced during liver dissociation performed to isolate hepatocytes (not shown). In contrast, no significant increase in caspase 3 activity and TUNEL staining (not shown) were observed after PH.

In conclusion, although many reports previously evidenced the regulation of signaling pathways by GSTs in various *in vitro* cell systems, we demonstrate for the first time that the invalidation of *Gstp1/p2* affects multiple key events of the hepatocyte cell cycle *in vivo* after PH. Accumulation of lagging events immediately after PH and throughout the progression in G1, including the alteration of the finely tuned activations of JNK and ERK1/2, considerably delays the commitment to DNA replication and mitosis.

## Materials and methods

### Animal experiments

All experiments complied with ethical laws, and experimental procedures were approved by ethical committee (no. R-2010-AC-01). C57BL6/J WT (Gstp1/2^+/+^) mice and Gstp1/2 knockout (Gstp1/2^*−/−*^) mice in C57BL6/J background were provided by Professor Roland Wolf and Cancer Research UK.^25^ Animals were maintained on a standard diet and housed under a 12-h light/dark cycle. Adult male mice (8- and 12-weeks old) were subjected to 70% PH under ketamine/xylazine anesthesia. Briefly, mice were subjected to mid-ventral laparotomy, and resection of right medial, left medial and left lateral lobes were performed after separate ligation. A special care was taken to preserve the gallbladder. Remnant livers (right lateral and caudal lobes) and blood samples (collected from retro-orbital sinus under anesthesia before killing) from three to seven mice per genotype were harvested at various time points from 30 min to 7 days.

### Liver function, histology, BrdU labeling and immunohistochemistry

Serum levels of triglycerides, ALT, AST, bilirubin and urea were measured using an automatic analyzer (Olympus automated chemistry Analyzer AU 2700; Olympus France, Rungis, France) following the manufacturer's instruction.

Hematoxylin–eosin-stained paraffin sections were performed for liver histology evaluation. Detection of GSTP1/2, NRF2 and MnSOD were performed on paraffin sections by using anti-GSTpi (PB13020, Abnova, Tebu Bio Sas, Le Perray en Yvelines, France), anti-NRF2 (sc-722, Santa Cruz Biotechnology, Santa Cruz, CA, USA) and anti-MnSOD (D3X8F, Cell Signaling Technology, Beverly, MA, USA), respectively.

To measure DNA replication, a single intra-peritoneal injection of BrdU (Sigma-Aldrich, Saint-Quentin Fallavier, France; 0.5 mg/g animal weight) was realized 1 h before killing. Livers were fixed, and cells in S or M phases were then detected by using anti-BrdU (ab8152, Abcam, Cambridge, UK) or anti-phospho-histone H3 (no. 9701, Cell Signaling Technology), respectively. A co-staining for nucleus was performed with Hoechst. Cell counting was made with the Simple PCI software (Hamamatsu Photonics, Massy, France) on × 20 pictures with at least 500 cells.

### Caspase 3 activity assay

Fifty milligrams of frozen remnants livers were lysed in caspase 3 activity buffer (20 mM PIPES, 100 mM NaCl, 10 mM DTT, 1 mM EDTA, 0.1% CHAPS, 10% sucrose, pH 7.2). One hundred micrograms of proteins were incubated with 100 *μ*M DEVD-AMC at 37 °C for 1 h. The liberation of AMC was monitored using excitation/emission wavelength of 380/440 nm (Gemini, Molecular Devices, Saint Grégoire, France). Caspase 3 activity was measured as arbitrary fluorescent units.

### Western blotting analysis and enzyme-linked immunosorbent assay (ELISA) assay

Whole liver lysates were prepared by sonication in HEPES lysis buffer (50 mM HEPES pH 7.5, 150 mM NaCl, 1 mM EDTA, 2.5 mM EGTA, 0.1% Tween 20, 10% glycerol, 0.1 mM Na orthovanadate, 1 mM NaF, 10 mM β-glycerophosphate) containing protease inhibitor cocktail (EDTA-free Complete Mini, Roche, Meylan, France). Pool of total proteins extracted from three livers at each time point were performed, and proteins were separated using the NuPAGE Novex Bis-Tris 4–12% Gels Kit (Invitrogen, Saint Aubin, France). Western blottings were performed according to the standard procedure using the following antibodies: anti-GSTpi (Abnova, PB13020); anti-JNK (no. 9258), anti-phosphoJNK (no. 4671), anti-STAT3 (no. 9132), anti-phospho-STAT3 (no. 9131), anti-ATF2 (no. 9226), anti-phospho-ATF2 (no. 5112), anti-p27^Kip1^ (no. 2552), anti-p38 (no. 9212), anti-phosphop-38 (no. 4511), anti-ERK (no. 9102), anti-phospho-ERK (no. 9101) and anti-phospho-c-Jun (no. 9261) from Cell Signaling Technology; anti-c-Jun (sc-1694), anti-CDK2 (sc-163), anti-CDK4 (sc-260), anti-Cyclin E (sc-25303), anti-MCM7 (sc-9966), anti-p53 (sc-6243), anti-TRAF2 (sc-876), anti-HO-1 (sc-136960), anti-Nrf2 (sc-722), anti-HSC70 (sc-7298) and anti-p21^Cip1^ (sc-397) from Santa Cruz Biotechnology; anti-iNOS (610431) and anti-eNOS (610298) from BD Transduction Laboratories, BD Biosciences, Le Pont de Claix, France; anti-E2F1 (OABB00513) from Aviva Systems Biology (CliniSciences Nanterre, France), anti-catalase (AF3398) from R&D Systems Europe (Lille, France); and anti-MnSOD (D3X8F) from Cell Signaling Technology. Protein expression was visualized using an Enhanced Chemiluminescence Kit (ECF; Amersham Biosciences, Orsay, Fance), and the signals were quantified with scanning densitometry using the Quantity One software program (Bio-Rad Laboratories, Hercules, CA, USA). Densitometry data were normalized with the loading control HSC70 for Cyclin D1 and CDK1, total ERK for p-ERK and JNK for p-JNK.

For ELISA, liver tissues were sonicated in 50 mM Tris-HCl pH 7.5, 150 mM NaCl, 1% Triton X-100 and protease inhibitor cocktail (EDTA-free Complete Mini, Roche). IL-6 and TNF*α* levels were determined by the commercial ELISA assay (DY406 and DY410, respectively, R&D Systems Europe) according to the manufacturer's guidelines.

### Real-time PCR

Total RNA from remnants livers (*n*=3–7 by time point) were extracted with RNeasy Mini Kit (QIAGEN, Courtaboeuf, France; Cat. No. 74106) following the manufacturer's instructions. Reverse transcription was performed using 1 *μ*g of total RNA with the High Capacity cDNA Reverse Transcription Kit (Applied Biosystems, Part No. 4368813). mRNA levels of various genes was determined using Taqman- or SYBR green-based quantitative PCR (qPCR) technology performed in 7900HT system (Applied Biosystems), and TBP was used as a housekeeping gene.^43^ Primers are listed in Tables 1 and 2.

### Statistical analysis

Results are presented as mean±S.E.M. Statistical analyses were performed with the GraphPad 5.0 software (La Jolla, CA, USA) with ANOVA non-parametrical test and then unpaired Student's *t*-test.

## Figures and Tables

**Table 1:** List of the Taqman primers

**Gene**	**Applied denomination**	**Reference**
18S	RN18S	Mm03928990-g1
Cdk1 (cyclin-dependent kinase 1)	Cdc2a	Mm00772472-m1
c-Fos	Fos	Mm00487425-m1
c-Jun	Jun	Mm00495062-m1
c-Myc	Myc	Mm00487804-m1
Cyclin D1	Ccnd1	Mm00432359-m1
HGF (hepatocyte growth factor)	Hgf	Mm01135193-m1
JunB	Junb	Mm01251660-m1
p53	Trp53	Mm01731287-m1
TBP (TATA-binding protein)	TBP	Mm00446971-m1
Gstp1 (glutathione transferase P1)	Gstp1	Mm00496606-m1
Gstp2 (glutathione transferase P2)	Gstp2	Mm01231544-m1

**Table 2:** List of the SYBR green primers

	**Forward primer (5′→3′)**	**Reverse primer (5′→3′)**
mMnSOD (manganese superoxide dismutase)	TGGTGGTCCATGAGAAACAA	GTTTACTGCGCAATCCCAAT
mHsp70 (heat-shock protein)	TATGCCTTCAACATGAAGAGCGCC	CTTGTCCAGCACCTTCTTCTTGTC
mHmox-1 (heme-oxygenase 1)	CACGCATATACCCGCTACCT	CCAGAGTGTTCATTCGAGCA
mNrf2 (nuclear factor erythroid 2-related factor)	AGGACATGGAGCAAGTTTGC	TCTGTCAGTGTGGCTTCTGG
mTBP (TATA-binding protein)	AGGGGCAATGTAACACAGGT	GGTGCATCGAGTCCGGTA
mHif1*α* (hypoxia-inducible factor 1*α*)	GTCACCTGGYYGCTGCAATA	CATGATGGCTCCCTTTTTCA
mCAT (catalase)	TGAGAAGCCTAAGAACGCAATTC	CCCTTCGCAGCCATGTG
mNOSi (inducible NO synthase)	AACGGAGAACGTTGGATTTG	CAGCACAAGGGGTTTTCTTC
mNOSe (endothelial NO synthase)	CCTTCCGCTACCAGCCAGA	CAGAGATCTTCACTGCATTGGCTA

## Abbreviations

PH: partial hepatectomy
GST: glutathione *S-*transferase
GSH: glutathione
TRAF2: tumor necrosis factor receptor-associated factor 2
ASK1: apoptosis signal regulating kinase 1
MAPK: mitogen-activated protein kinase
JNK: c-jun N-terminal kinase
ERK: extracellular signal regulated kinase
MCM: minichromosome maintenance
ROS: reactive oxygen species
BrdU: 5-bromo-2'-deoxyuridine
ATF: activating transcription factor
CDK: cyclin-dependent kinase
ALT: alanine aminotransferase
AST: aspartate aminotransferase
